# A novel lifting point location optimization method of transmission line tower based on improved grey wolf optimizer

**DOI:** 10.1038/s41598-023-49043-y

**Published:** 2023-12-08

**Authors:** Guolin Wang, Pengjie Ding, Chaosheng Huang, Zhongliang Yu

**Affiliations:** Zhejiang Electric Transmission and Transformation Co., Ltd., Hangzhou, 310000 China

**Keywords:** Engineering, Mathematics and computing

## Abstract

In the erection process of transmission line tower, the appropriate lifting point position is an important factor in ensuring the stability and balance of the lifting process and preventing deformation and damage to the towers. In this paper, a improved grey wolf optimization algorithm is proposed to solve the issues of low optimization efficiency and easily getting trapped in local minima when optimizing the lifting point position of transmission line towers. The improved algorithm includes the use of a good point-set strategy to enhance the initialization method of grey wolf individuals, ensuring a more uniform distribution of the population and reducing ineffective searches in the early stages of optimization. Furthermore, two random operators are utilized to combine and mutate the optimal grey wolf position, thereby enhancing the algorithm's ability to escape local optima. Finally, the trend information of the optimization process is considered, and the median value of the population is used to improve the stability of the optimization algorithm. Experimental results demonstrate that the proposed algorithm has better optimization performance and faster convergence speed compared to genetic algorithm, particle swarm optimization algorithm, and artificial fish swarm algorithm. It effectively addresses the optimization problem of lifting point position for transmission line towers.

## Introduction

Ultra-high voltage (UHV) power transmission has the advantages of less pollution, lower loss, and higher benefit, which intersects with other energy transmission modes and has greater demand^1^, and transmission line tower is an important equipment used in it^2^. In order to meet the increasing needs of transmission line tower erection and ensure the high efficiency, stability, and safety of transmission line tower erection, it has become an important research direction in transmission line tower erection on how to choose the lifting point. Scientifically choosing the four-point lifting of transmission line tower can ensure the stable balance of the process, and make the transmission line tower bear uniform force to prevent excessive deformation and damage^3^.

The selection of lifting point positions for transmission towers can be seen as an optimization problem. Currently, many experts and scholars have conducted research on optimization problems in such projects, aiming to achieve the project objectives by using swarm intelligence optimization algorithms and making improvements. These algorithms can effectively solve higher-dimensional global optimization problems and handle multiple local optima simultaneously. These characteristics make these algorithms popular and widely used for solving global optimization problems^4^. However, there is no universally applicable optimization algorithm that can solve all optimization problems, so algorithms need to be modified and optimized to adapt to new engineering problems^5^. Novel methods have been proposed in Refs.^6–9^ to address the issues in the standard Whale Optimization Algorithm (WOA). The improved algorithms can be applied to large-scale engineering optimization problems, improving the accuracy and diversity of the algorithms and avoiding premature convergence issues. A variant of the Butterfly Optimization Algorithm with a self-adaptive parameter setting, Lagrange interpolation formula, and a new local search strategy embedded with Levy flight search is proposed in Ref.^10^. This variant enhances the searching ability of the algorithm, achieving a better trade-off between exploration and exploitation. Research has been conducted on the initialization methods of swarm intelligence optimization algorithms in Refs.^11–13^, optimizing the optimization effectiveness and convergence of the algorithms. In practical engineering applications, Ref.^14^ successfully reduced costs by using a genetic algorithm to optimize the positions of cranes and trailers during the lifting process of heavy modularized building units. Reference^15^ optimized the lifting sequence using the A-star algorithm, ensuring engineering safety and reducing costs. Based on genetic algorithms and ant colony algorithms, Refs.^16–18^ planned the lifting paths of cranes in complex environments to avoid collisions. Improvements have been made to the particle swarm optimization algorithm in Refs.^19–21^ and applied to optimize the lattice structure of transmission towers, ensuring structural stability and reducing material consumption. An improved genetic algorithm is utilized in Ref.^22^ for the optimization design of transmission tower structures, achieving a 10% reduction in tower costs. Modifications to the Jaya algorithm were proposed in Refs.^23,24^, reducing the number of parameters and enhancing the optimization effectiveness of the lattice size and layout of transmission towers. In Ref.^25^, a COVID-19 X-ray image segmentation method based on improved WOA achieved good segmentation results.

Overall, swarm intelligence optimization algorithms have been widely applied to relevant engineering optimization problems. The Grey Wolf Optimization (GWO) algorithm, with its simplicity, wide applicability, and few adjustable parameters, is the preferred solution for highly nonlinear and multivariable problems like optimizing the lifting positions of transmission towers^26^. Reference^27^ applied GWO to unmanned aerial vehicle path planning tasks, and simulation results showed that the proposed method outperformed other state-of-the-art algorithms in terms of quality, speed, and stability. Reference^28^ proposed a hybrid GWO based on elite opposition to improve the population diversity and convergence speed of the algorithm. Experimental results using multiple benchmark functions showed that the algorithm had strong global and local search capabilities, fast convergence speed, and high accuracy. Reference^29^ combined GWO with lateral inhibition to solve complex template matching problems, and compared with other lateral inhibition-based algorithms, this method achieved the best balance between estimation accuracy and computational cost.

The optimization of lifting positions for transmission towers with four lifting points has similarities to the aforementioned engineering problems. However, the standard GWO algorithm often fails to achieve satisfactory results for optimizing the lifting positions in such scenarios. Furthermore, there is limited research on improvement methods for optimizing the lifting positions of transmission towers with four lifting points. Therefore, this paper addresses the shortcomings of the current application of the GWO algorithm to optimize the lifting positions of transmission towers with four lifting points. Optimization is performed on the initialization and iteration strategies of GWO, and experimental results demonstrate that the proposed improved GWO outperforms other optimization algorithms.

## Optimization of lifting location of transmission line tower based on GWO

### Optimization model of lifting point based on energy principle

In the calculation of finite element structure, we cannot only take the maximum stress of the whole structure as the judging condition of the design of lifting point because the structure itself or different modeling methods will have the phenomenon of stress concentration. Taking the minimum strain energy of the whole structure as the goal, the allowable load rate of crane is taken as the constraint condition to construct the optimization model.

Energy principle is widely used in the field of mechanics and finite element analysis. Strain energy as a state variable characterizes the stress state of structures. This method has been widely used in engineering, and has achieved good results in the construction of long-span cable-stayed bridges, concrete construction and lifting.

In elastic materials, omitting the energy consumption generated during loading and unloading of external forces, the work done by external forces is numerically equal to the strain energy $U$ stored in the structure during this process. For structures bearing axial loads, assuming that the axial stiffness $EA_{i}$ and internal force $N_{i}$ on the members are within the linear elastic range, then:

1 $$U = \frac{1}{2E}\sum\limits_{i = 1}^{n} {\frac{{N_{i}^{2} l_{i} }}{{A_{i} }}}$$

Derive $$N_{i}$$, then

2 $$\frac{\partial U}{{\partial N_{i} }} = \frac{{N_{i} }}{{EA_{i} }}$$

It can be seen from the above equation that the total strain energy of the structure and the stress of the members have the same monotonicity, so the quantitative index of the minimum strain energy of the whole structure is taken as the standard to judge the rationality of the design of the lifting point system.

The optimization model of four-point lifting location can be expressed as Eq. (3):

3 $$\begin{aligned} & Min\quad E(x,y) = \frac{1}{2E}\sum\limits_{i = 1}^{n} {\frac{{N_{i}^{2} l_{i} }}{{A_{i} }}} \\ & s.t.\quad F_{i} (x,y) - [F_{i} ] \le 0 \\ & \quad 1 \le Num_{i} (y) \le n,i = 1, \ldots ,x \\ \end{aligned}$$

Among them, $x$ is the quantity of lifting points, $y$ is the location of lifting points, $F_{i} (x,y)$ is the actual stress of crane, $[F_{i} ]$ is the allowable load of crane, $E(x,y)$ is the overall strain energy of structure and $Num_{i} (y)$ is the number of lifting points.

The fitness function constructed for the mathematical model established by the above equation is shown in Eq. (4):

4 $$\begin{aligned} {\text{eval}}(x,y) & = E(x,y) + s\phi (x) \\ & \quad + r\mathop {\sum\limits_{k = 1}^{{N_{c} }} {\max [0,F_{i} (x,y) - [F_{i} ]]^{2} } }\limits^{.} \\ \end{aligned}$$

The penalty function is established according to the outer penalty function, which ${\text{eval}}(x,y)$ is fitness function, $r$ is constraint penalty factor, $s$ is discrete penalty factor and $\phi (x)$ is discrete penalty function. Because the number of lifting points is fixed at 4 and the locations of lifting points are all positive integer numbers, the discrete penalty term $s\phi (x)$ can be set to 0 to improve the optimization efficiency.

### Grey wolf optimizer

**Figure 1:**
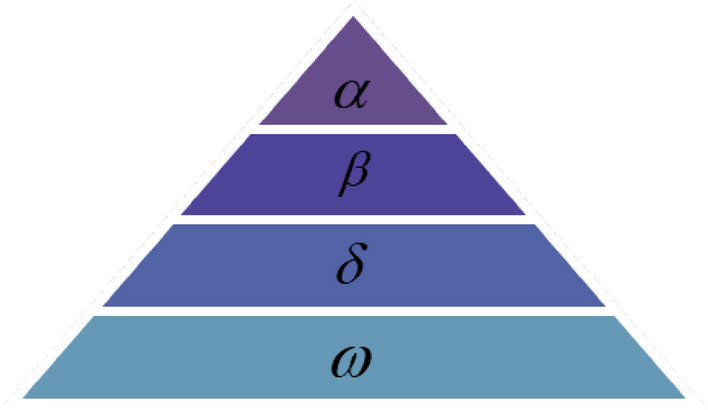
Gray wolf population hierarchy.

GWO is an optimization algorithm that imitates grey wolf hunting and social hierarchy behavior, which has the characteristics of strong convergence performance, few parameters and easy implementation. In the GWO, wolves are divided into four levels $\alpha ,\beta ,\delta ,\omega$, and the high-level gray wolves lead the low-level wolves to move in the optimal direction of the search space. Its social hierarchy is shown in Fig. 1.

**Figure 2:**
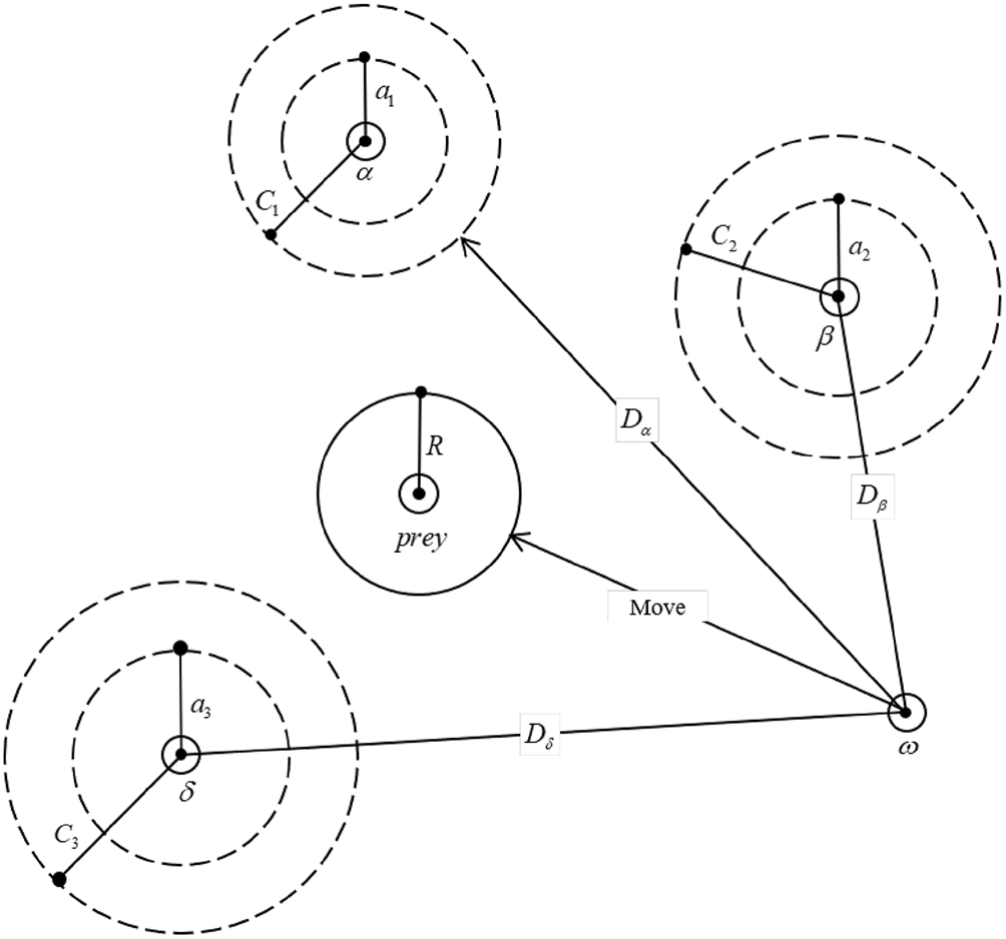
Searching process of gray wolf population.

GWO simulates the hunting behavior of gray wolves, so as to get a better solution. The search process of the GWO is shown in Fig. 2.

The optimization process of GWO is as follows:

Step 1 Initialization parameters, it is necessary to set the population size $n$, dimension $d$, maximum iteration time $T$ and convergence factor $a = 2 - 2t/T$ in the GWO, and randomly initialize the initial position of the gray wolf population:
  5 $$X_{i} ,(i = 1,2, \ldots ,n)$$
Step 2 Bring the individual position $X_{i}$ of wolves into the Fitness Function to find the fitness $F_{i} = Fitness(X_{i} )$ of all gray wolves, and take the best three individuals as the first wolves $X_{\alpha } ,X_{\beta } ,X_{\delta }$.
Step 3 Calculate the coefficient vector $A$ and $C$ as shown in Eq. (6):
  6 $$\begin{aligned} & A_{l} = 2a \cdot r_{1} - a \\ & C_{l} = 2 \cdot r_{2} \\ \end{aligned}$$
  $r_{1}$ and $r_{2}$ are random vectors with modulo 1, $l = \{ \alpha ,\beta ,\delta \}$
Step 4 Recalculate the position of the gray wolf, first calculate the distance between the gray wolf and the head wolf, as in Eq. (7):
  7 $$D_{l} = \left| {C_{l} \cdot X_{l} - X} \right|$$
  $X_{l}$ and $X$ are the positions of the head wolf and the current gray wolf individual respectively. Then update the position according to the distance between the current gray wolf individual and each wolf:
  8 $$X = \frac{{\sum\nolimits_{l = \alpha ,\beta ,\delta } {X_{l} - A_{l} \cdot D_{l} } }}{3}$$
Step 5 Terminate the condition and judge whether the maximum iteration times are reached, otherwise jump to Step 2 to continue iterative optimization. Terminate the algorithm process and output the optimal result achieved by gray wolf individuals in the search process.

### Simulation of GWO

Four kinds of optimization test functions with multiple local minima are used to simulate the particle swarm optimization algorithm for test. The functions used are Salomon Function:

9 $$f(x) = 1 - \cos \left( {2\pi \sqrt {\sum\limits_{i = 1}^{n} {x_{i}^{2} } } } \right) + 0.1\sqrt {\sum\limits_{i = 1}^{n} {x_{i}^{2} } }$$

**Figure 3:**
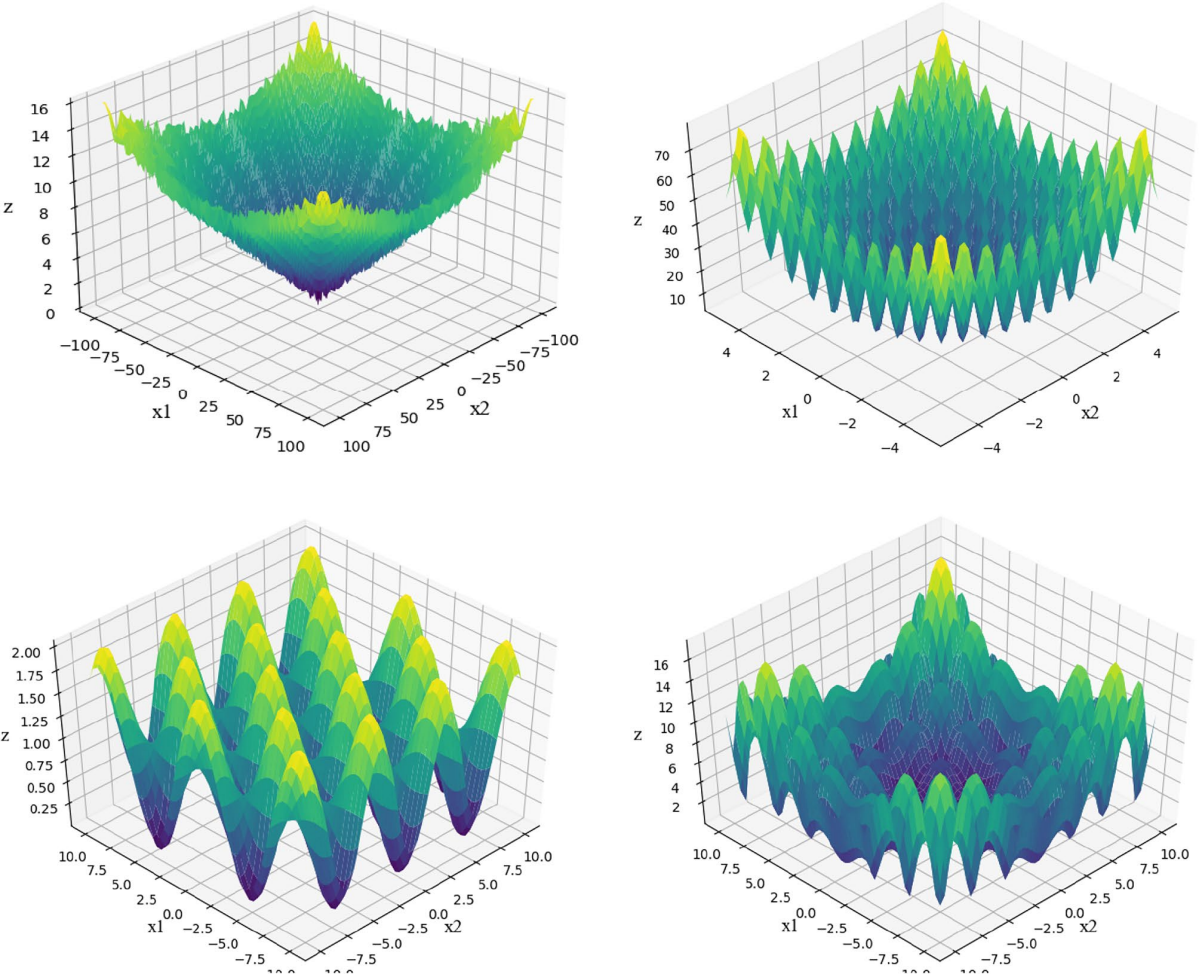
Images of four test functions.

- Rastrigin Function:
  10 $$f_{2} (x) = 10d + \sum\limits_{i = 1}^{d} {[x_{i}^{2} - 10\cos (2\pi x_{i} )]}$$
- Griewank Function:
  11 $$f_{3} (x) = \sum\limits_{i = 1}^{d} {\frac{{x_{i}^{2} }}{4000} - \prod\limits_{i = 1}^{d} {\cos \left( {\frac{{x_{i} }}{\sqrt i }} \right)} + 1}$$
  Alpine Function:
  12 $$f_{4} (x) = \sum\limits_{i = 1}^{d} {\left| {x_{i} \sin (x_{i} ) + 0.1x_{i} } \right|}$$
  where $d$ is the dimension of parameters in the function, the image of the above test function in the parameter dimension $d = 2$ is shown in Fig. 3.

**Figure 4:**
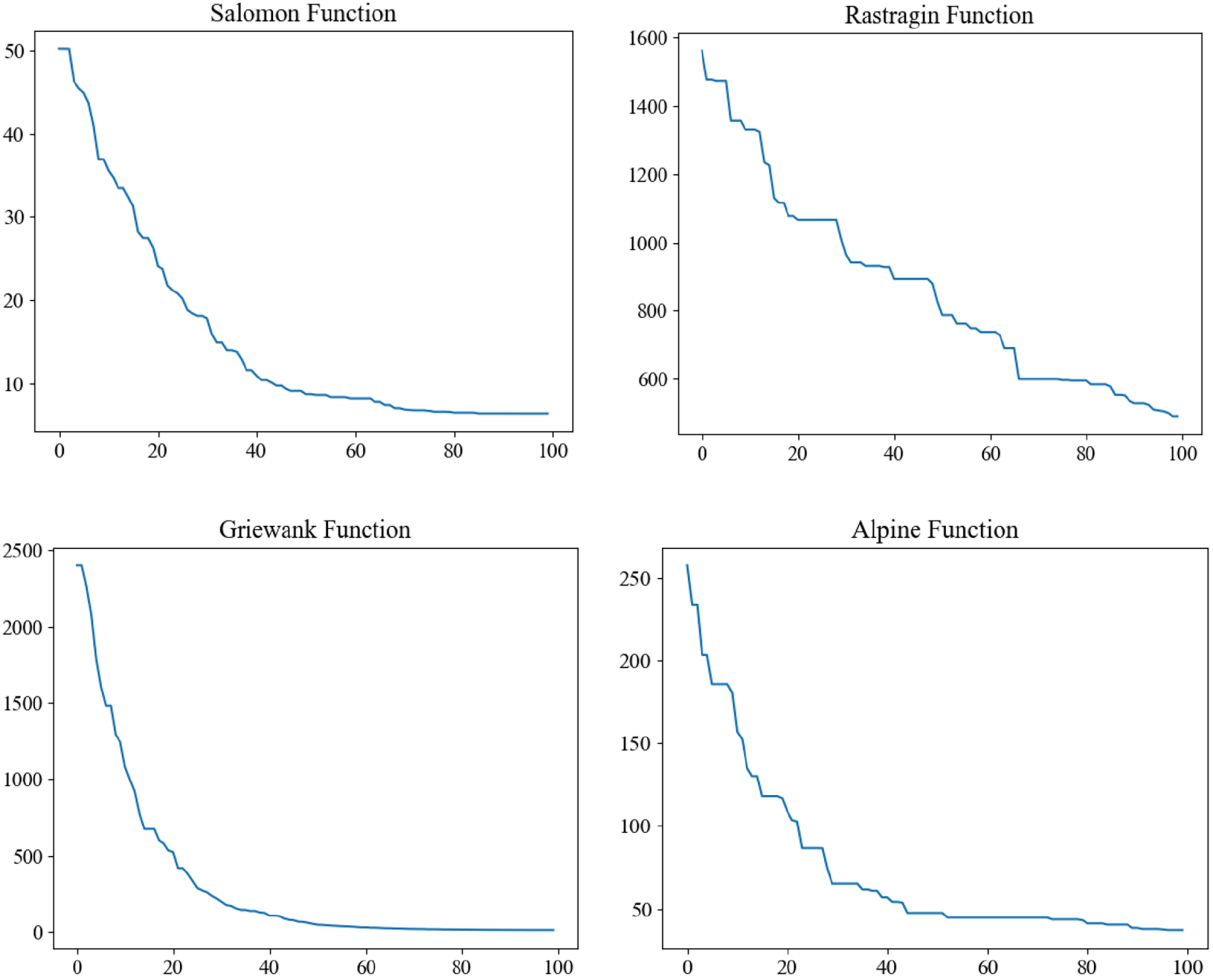
Optimal iterative curves of four test functions.

All the above three test functions have global minimums $f(x*) = 0$ and the minimum points are located at the origin, that is, $x* = (0, \ldots ,0)$. Set the parameter dimension $d = 100$, population size $I = 10$ and maximum iteration number $T = 100$ of test function and carry out simulation experiments. The optimization curves obtained by four test functions using GWO are shown in Fig. 4.

It can be seen from the results in Fig. 4 that although the GWO can solve the optimization problem, the optimization efficiency is average when the number of gray wolves is low, and it is easy to fall into premature phenomenon prematurely, resulting in falling into local optimum.

## Improvement of GWO

### Population initialization method based on good point-set

GWO adopts random initialization strategy, which would lead to blind initial solution, and the error of each optimization result is large. The individual of the initial solution cannot uniformly traverse the position in the space, which will lead to the absence of some better solutions. In addition, if the initial population is distributed near the local extremum, the local optimum value will be regarded as the global optimum value, which is a big defect for the algorithm. In this paper, a new initialization method is designed by using the good point-set^30^. If there is a unit cube $G_{t}$ and $r \in G_{t}$ in dimensional $D$ Euclidean space, there are:

13 $$P_{n} (k) = \{ r_{1}^{n} k, \ldots ,r_{2}^{n} k, \ldots ,r_{D}^{(n)} k, - 1 \le k \le n\}$$

If the above equation has a deviation as shown in Eq. (14), the set is called a good point-set, where $r$ is called a good point.

14 $$\phi (n) = C(r,\varepsilon )n^{ - 1 + \varepsilon }$$

where, $C(r,\varepsilon )$ is a constant, take $r_{k} = e^{k} ,1 \le k \le D$, $p$ is the minimum prime number satisfied $(p - D)/2 \ge D$.

**Figure 5:**
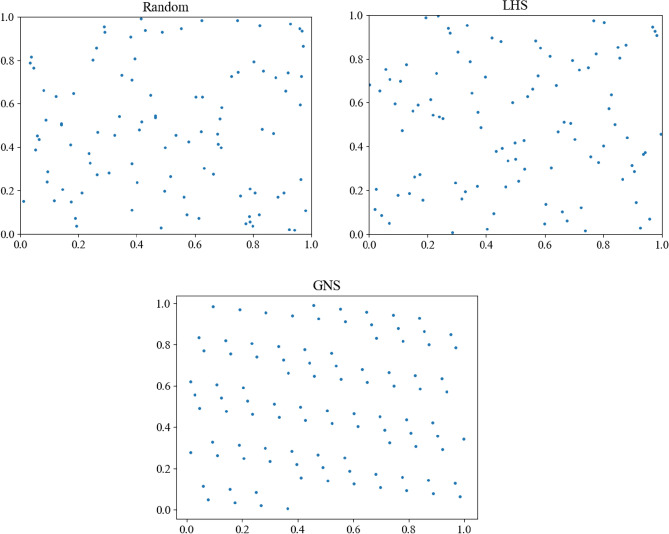
Comparison of three initialization methods in two-dimensional space.

In order to verify the superiority of this method, 100 points with values in the range $[0,1]$ are selected, and three methods are used to initialize the population: random sampling, Latin hypercube sampling and good point-set construction. The contrast distribution map in two-dimensional space is shown in Fig. 5.

From the above experimental results, we can see that using the population initialization method based on good point-set can ensure that the initial position distribution of the population is more uniform, so that individuals can spread all over the whole search space to avoid invalid search, effectively avoid the algorithm falling into the local optimal value and stagnating, and improve the global convergence.

In summary, the pseudo code of the improved algorithm is presented in Table 1.

**Table 1:**
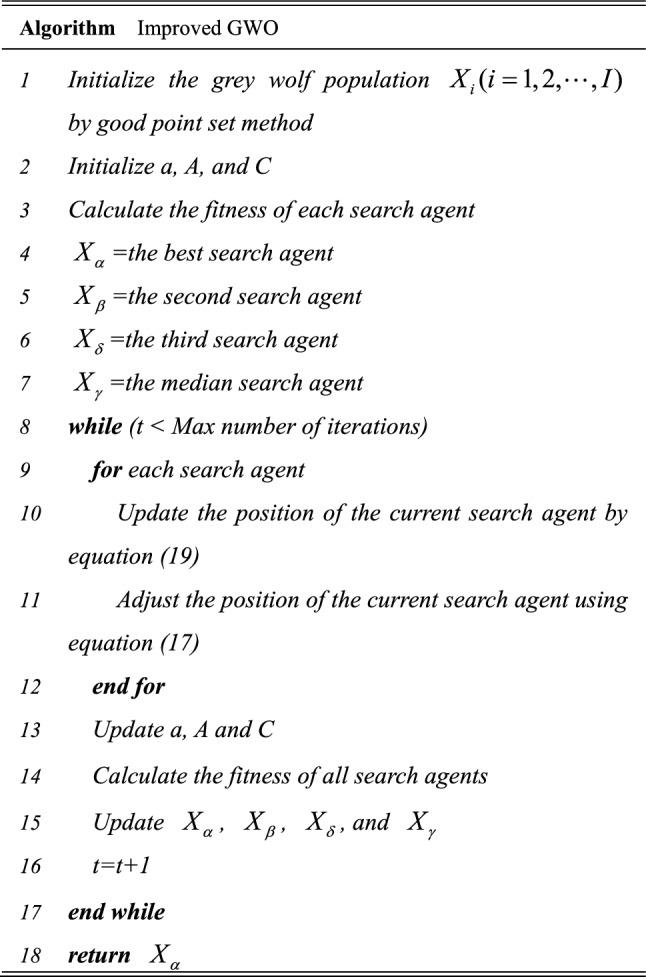
Pseudo code for the improved algorithm.

### Combination variation adjustment of optimal position

GWO is easy to fall into local optimum at the later stage of iteration, which leads to slow convergence and low convergence accuracy. In order to solve this problem, it is necessary to adjust the optimal position by mutation. In this paper, Cauchy random number is introduced into Gaussian mutation operator to disturb the GWO and improve the ability of the algorithm to jump out of local optimum.

Cauchy mutation operator is a random number based on Cauchy distribution and generated by Cauchy distribution function, as shown in Eq. (15):

15 $$y = \frac{1}{2} + \frac{1}{\pi }\arctan \left( {\frac{{x - x_{0} }}{\gamma }} \right)$$

Cauchy distribution is also called Cauchy–Lorentz distribution. Its density function is similar to Gaussian density function, but the speed approaching the central axis is very slow, and the variance is close to infinity. The probability density functions of Gaussian function and Cauchy function are shown in Eq. (16)

16 $$\begin{gathered} f_{g} (x;\mu ,\sigma ) = \frac{1}{{\sqrt {2\pi \sigma } }}e^{{ - \frac{{(x - \mu )^{2} }}{{2\sigma^{2} }}}} \hfill \\ f_{c} (\delta ;x_{0} ,\gamma ) = \frac{1}{{\pi \gamma \left[ {1 + \left( {\frac{{x - x_{0} }}{\gamma }} \right)^{2} } \right]}} \hfill \\ \end{gathered}$$

**Figure 6:**
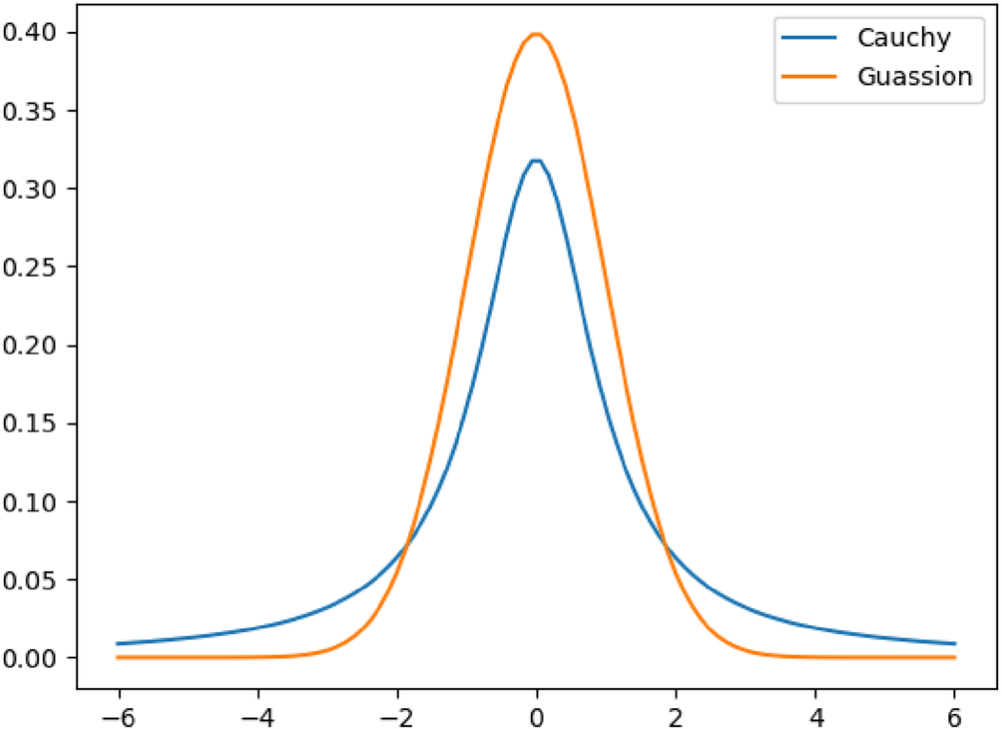
Standard Gaussian distribution function and standard Cauchy function.

The probability density functions of the standard Gaussian and Cauchy distributions are shown in Fig. 6.

When $x_{0} = 0$ and $\gamma = 1$, $f(\gamma )$ is the probability density function of standard Cauchy distribution. $y$ is a random number evenly distributed between $[0,1]$. Then the calculation method of Cauchy mutation operator can be obtained:

17 $$C(y) = \tan \left( {\pi \left( {y - \frac{1}{2}} \right)} \right)$$

**Figure 7:**
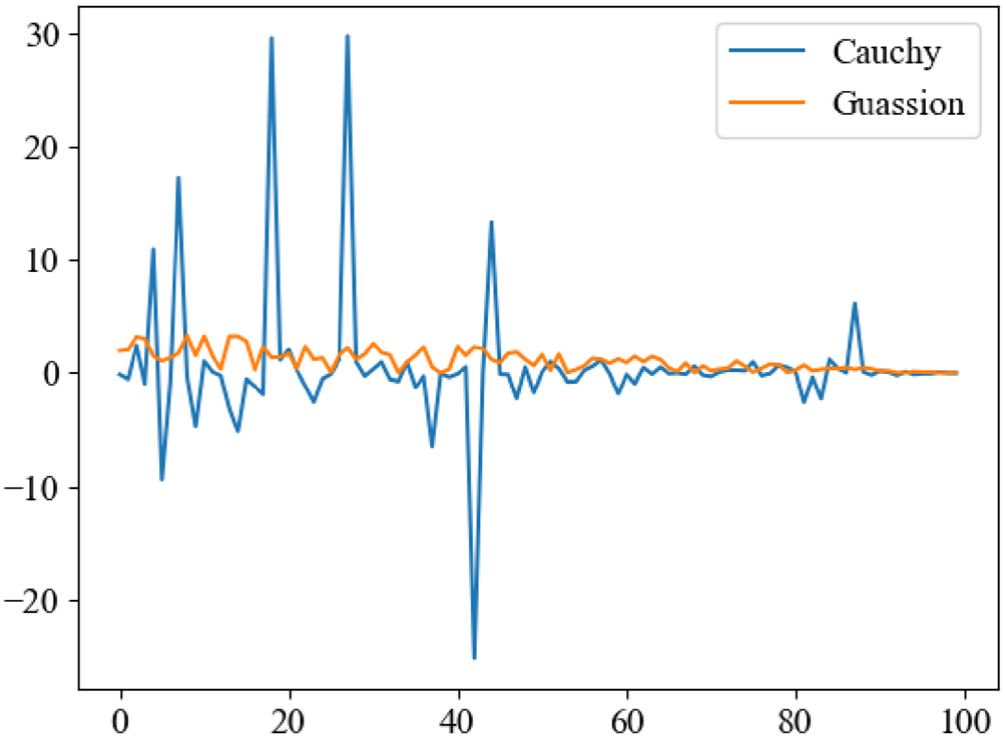
Generation effect of random numbers.

Combining the two operators for combined mutation, the optimal position of the GWO is disturbed, and the convergence factor $a$ is used to prevent the algorithm from being difficult to converge due to excessive disturbance in the later stage, as shown in Eq. (18):

18 $$X = X + a\frac{{G(r_{3} ) + C(r_{4} )}}{2}X$$

where $r$ is random numbers evenly distributed between $[0,1]$. The effect of this operator to generate random numbers under the influence of convergence factor $a$ is shown in Fig. 7.

The method makes full use of the advantages of Cauchy operator for fast convergence in large domain and Gaussian operator for small domain exploration. The perturbation can make the algorithm jump out of the current search range and find a new optimal value when it falls into local optimum again.

### Optimization strategy based on median

In the GWO, the best three individuals will be selected as the first wolf, and all other gray wolves will move according to the distance between them. It is precisely because of this characteristic that the diversity of grey wolf population is reduced, and the GWO is easy to fall into local optimum. This paper considers that when the GWO selects three wolves from the optimal position and it also sets one wolf at the median fitness of all gray wolves in the population, so as to introduce median information to optimize.

First, in Step 2, set a wolf in the median fitness of the gray wolf population and set it to $\gamma$:

19 $$\begin{aligned} & F_{\gamma } = {\text{median}}(F_{i} ) \\ & X_{\gamma } = X_{{{\text{index}}(F_{\gamma } )}} \\ \end{aligned}$$

The wolf also participates in the encirclement of prey. In hunting activities, the individual position update equation of gray wolf needs to consider the position of the wolf, that is, modify $l = \{ \alpha ,\beta ,\delta ,\gamma \}$.

Because the number of wolves guided by the optimal position is more than the median, in order to control the proportion of median information in the moving process of gray wolves, it is necessary to weigh it. Change the Grey Wolf Individual Position Update Equation:

20 $$X = (1 - \omega ) \cdot \frac{{\sum\nolimits_{\alpha ,\beta ,\delta } {X_{l} - A_{l} \cdot D_{l} } }}{3} + \omega (X_{\gamma } - A_{\gamma } \cdot D_{l} )$$

where $\omega$ is the scale factor of $[0,1]$, which is used to control the ratio between the median information and the optimal value. When setting $\omega = 0$, it is optimized by optimal value information completely, while $\omega = 1$ is optimized by median value information completely. In order to reduce the influence of median information in the later stage of the algorithm on optimization, convergence factor setting $\omega = a/2$ can be used.

The median position of gray wolf can take into account the action trend of all gray wolves, which is an index to reflect the group behavior. Through the above improvement of the GWO, the stability of the algorithm is enhanced, and the local optimum caused by excessive narrowing of the search range in the later stage of the algorithm is reduced.

## Simulation test and analysis

### Test function simulation and comparison of improved algorithm

**Figure 8:**
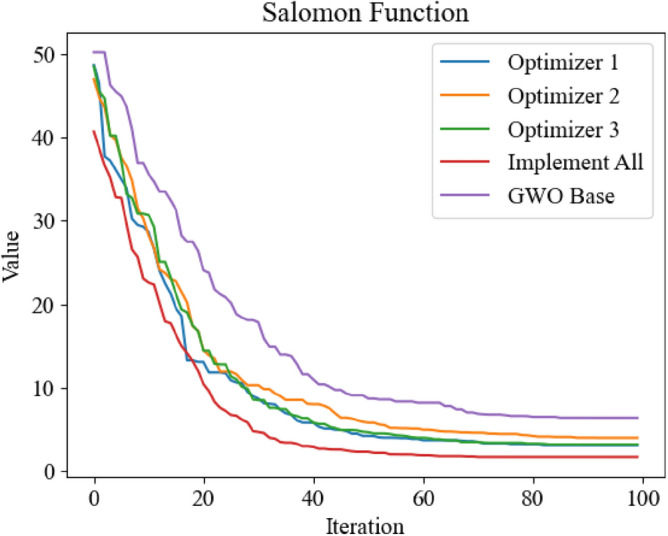
Optimization iteration curve of Salomon Function.

**Figure 9:**
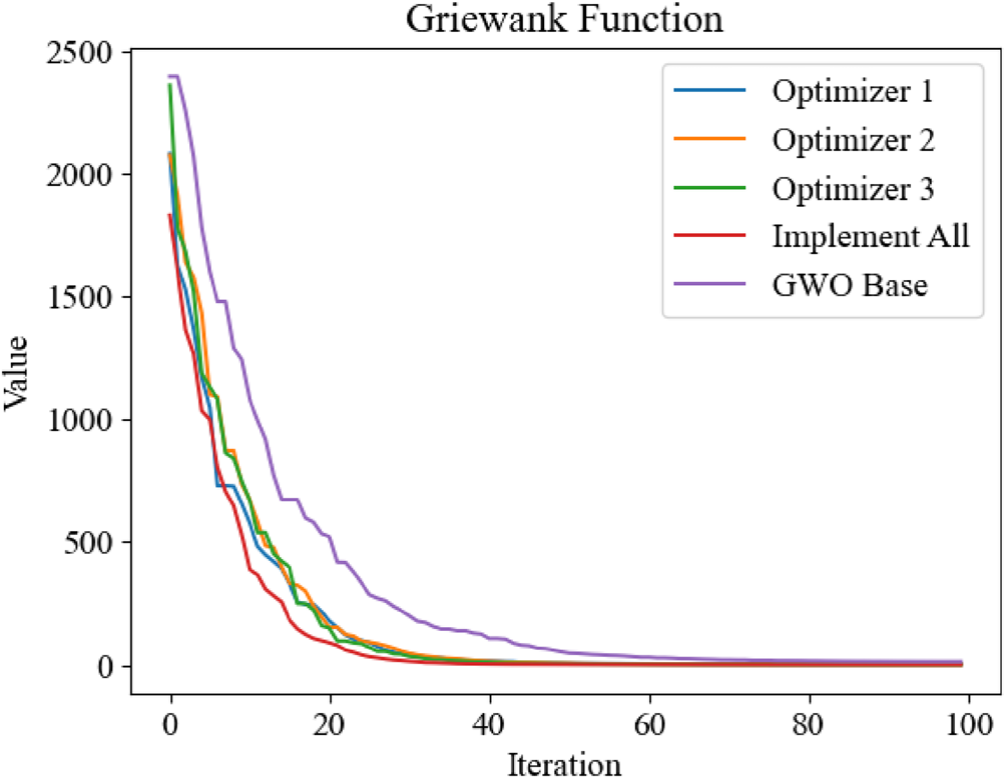
Optimization iteration curve of Griewank Function.

**Figure 10:**
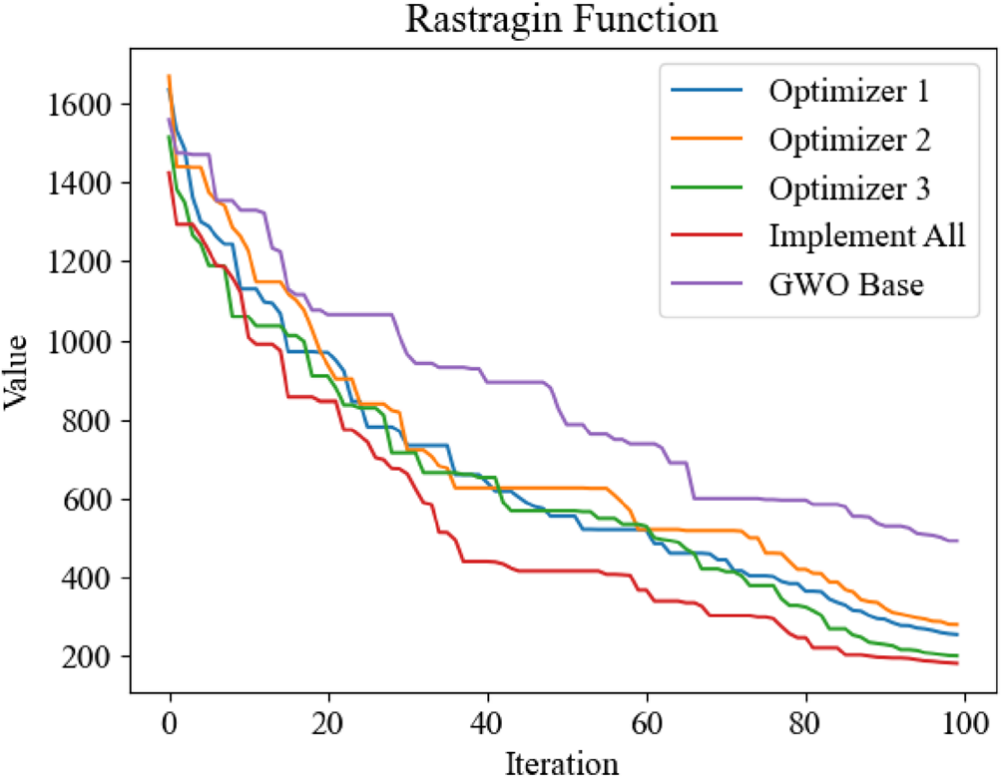
Optimization iteration curve of Rastragin Function.

1. In order to verify that the three improved methods proposed in this paper have practical improvement effect, firstly, the improved GWO is simulated by test function. The parameters of test function in 1.3 are optimized by using one of the three improved methods and all three improved methods, and compared with the standard GWO. The experimental results are shown in Figs. 8, 9, 10 and 11.

**Figure 11:**
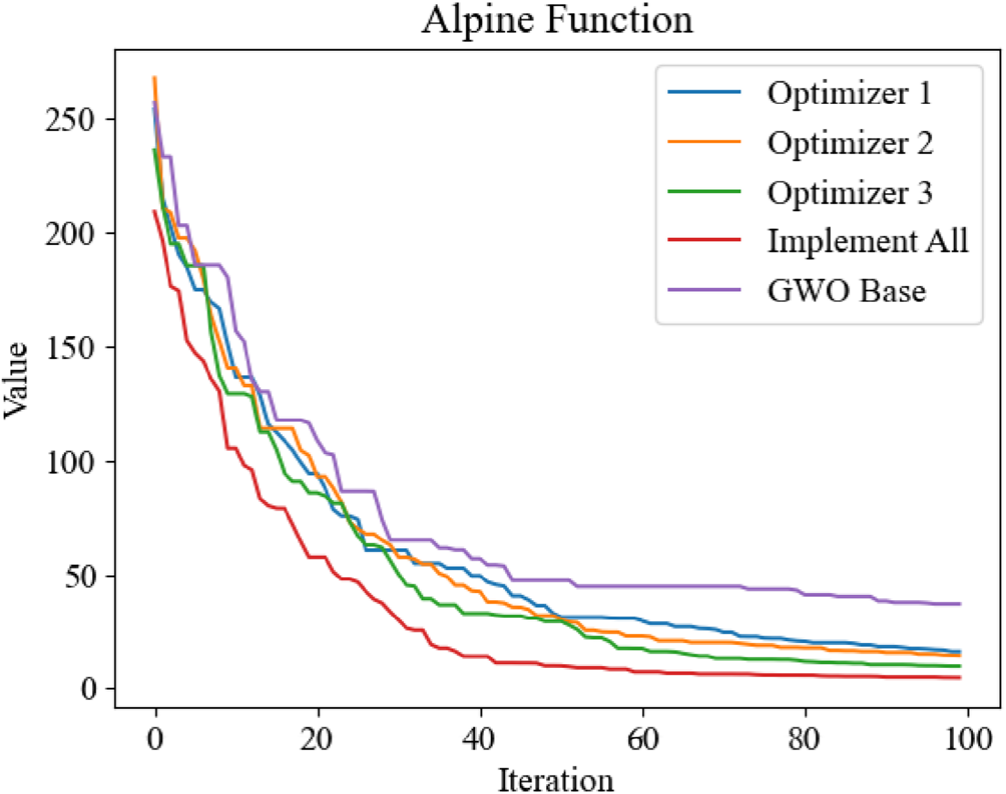
Optimization iteration curve of Alpine Function.
  It can be seen from Figs. 8, 10 and 11 that the three improved methods proposed in this paper can get better optimization results, which proves that the improved methods can improve the optimization ability of GWO. In Fig. 9, the standard GWO has achieved good results, so the improved GWO has little difference in final values, but it can be seen that the improved GWO can find the best point faster and realize fast convergence.
2. In order to further verify the superiority of the improved GWO, this paper also applies other swarm intelligence optimization algorithms to the above four test functions, and compares their simulation results with the improved GWO.
  The swarm intelligence optimization algorithms compared in simulation experiments include artificial fish swarm optimization algorithm, genetic optimization algorithm and particle swarm optimization algorithm. The algorithms participating in the comparison all initialize the positions of individuals by uniform distribution in the same range. The dimensions of test's function are all set to 100 dimensions, and the same number of iterations are carried out. The simulation experiments are carried out continuously for 10 times and the average of simulation results is taken to reduce errors. The experimental results are shown in Figs. 12, 13, 14 and 15.

**Figure 12:**
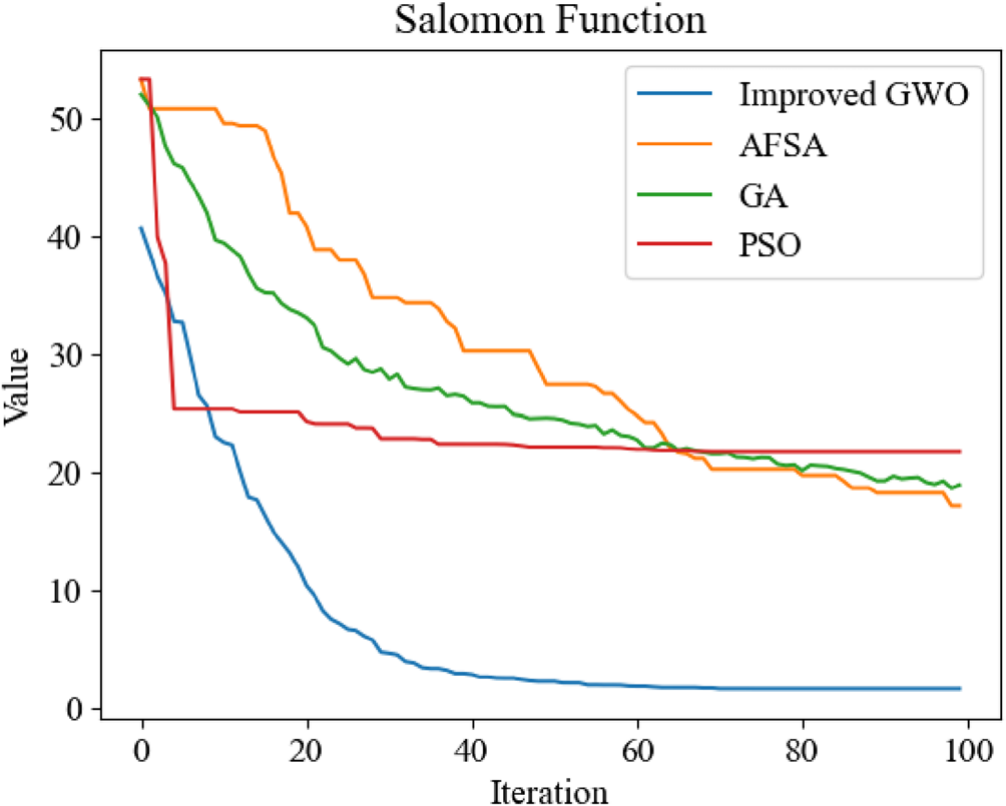
Comparison of optimization iteration curves for Salomon Function.

**Figure 13:**
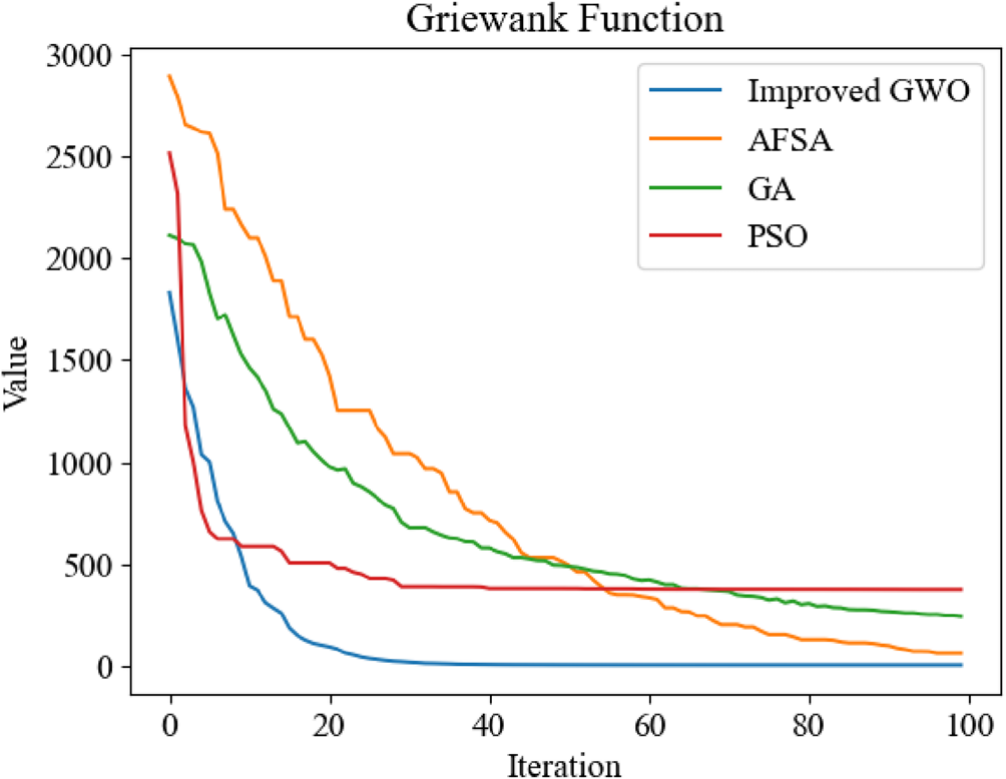
Comparison of optimization iteration curves of Griewank Function.

**Figure 14:**
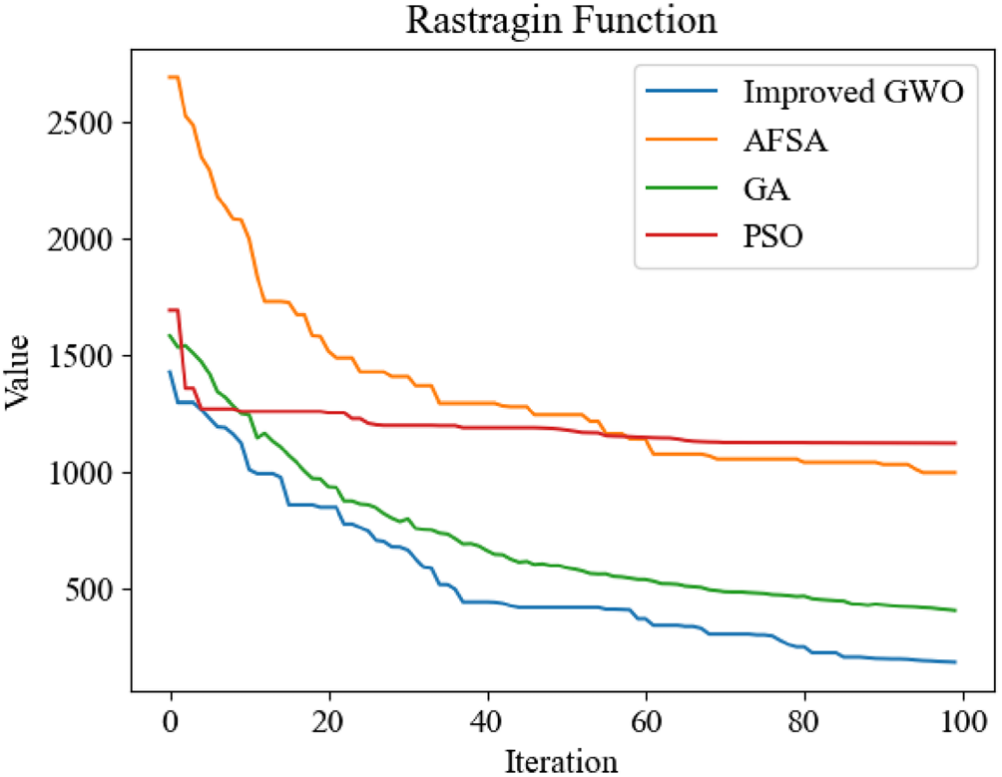
Comparison of optimization iteration curves of Rastragin Function.

**Figure 15:**
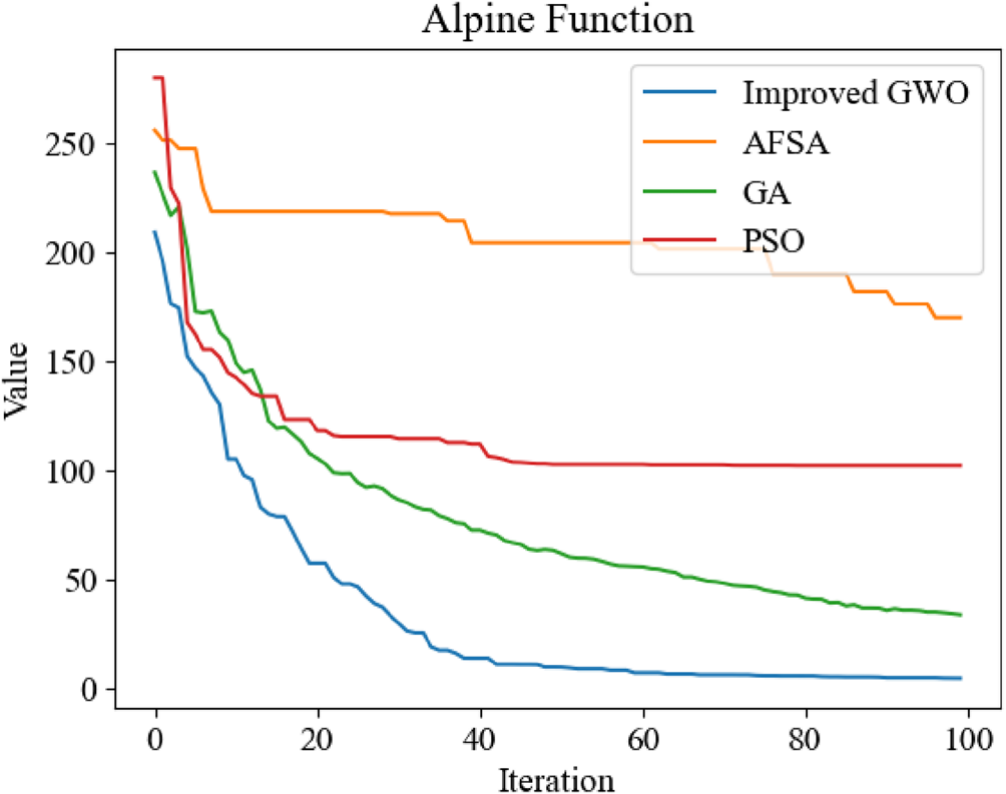
Comparison of optimization iteration curves for Alpine Function.
  It can be seen from Figs. 12, 13, 14 and 15 that the GWO after applying the improved method has faster optimization speed and is not easy to fall into local optimum in the above test function compared with other swarm intelligence optimization algorithms participating in test, and can get better optimization results.

### Simulation test for optimization of four-point lifting of transmission line tower

In order to verify the practical benefits of the improved algorithm, the improved GWO is used to optimize the four-point lifting of transmission line tower, and compared with other commonly used swarm intelligence optimization algorithms to reflect the superiority of the improved algorithm proposed in this paper.

**Figure 16:**
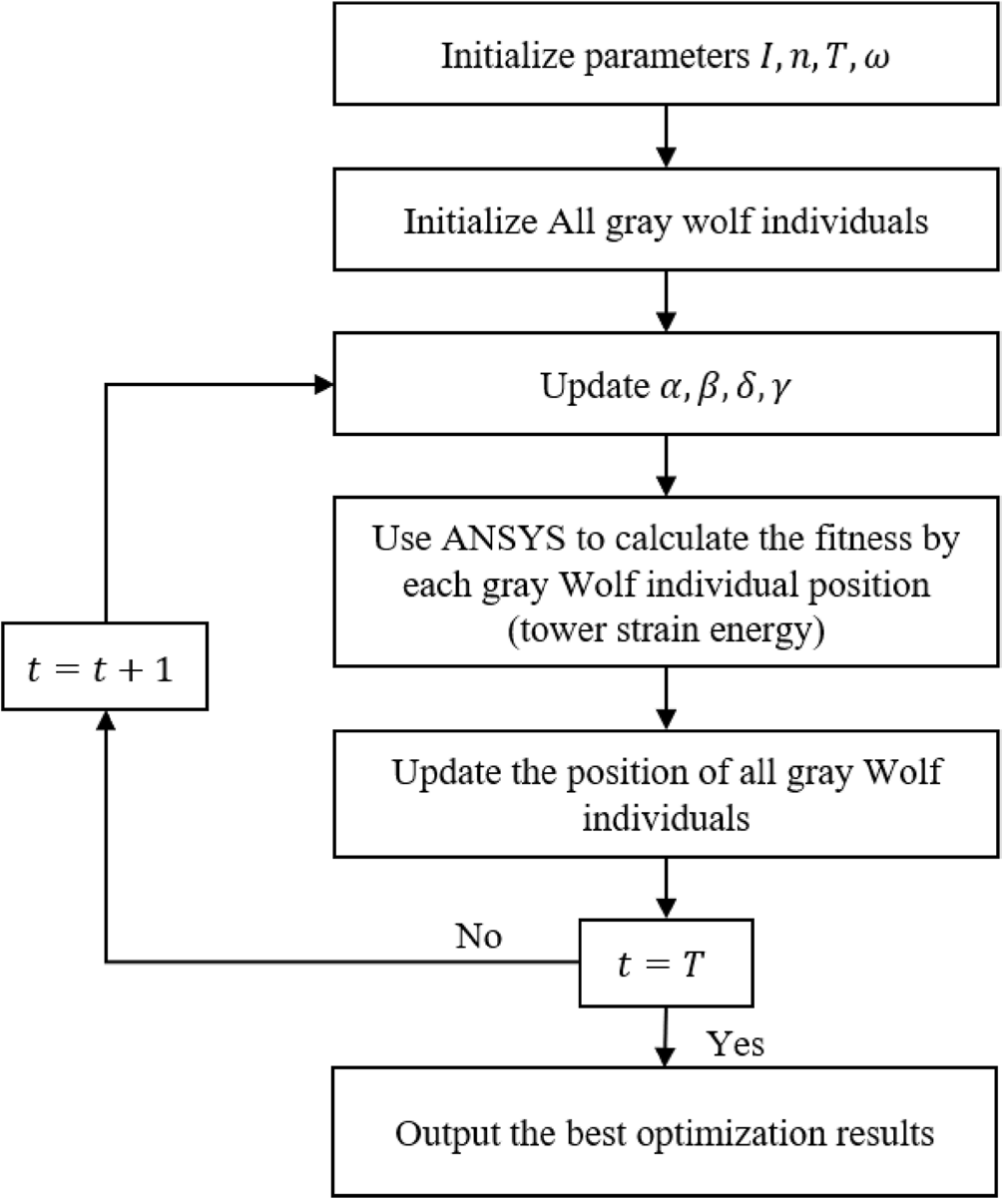
Optimization flow chart of four-point lifting of transmission line tower.

We conducted experiment using the ZB1V linear tower type as an example with the setting of population size $n = 10$, maximum number of iterations $T = 100$. By employing finite element modeling for transmission towers, the main material and significant secondary materials of the tower are simulated using the Beam188 beam element, while other secondary materials are simulated using the Link8 rod element. Formula (1) is used to calculate the strain energy of the transmission tower at different suspension points. Because the lifting point of the transmission line tower can only be located at the junction of the main girder, but not in the middle of the bar, the lifting location is discrete. The selection locations of different lifting points cannot be the same. It is necessary to ensure that the deformation and stress of the steel structure are minimized, that is, the overall strain energy of the corresponding steel structure is minimized. The optimization process of the lifting location is shown in Fig. 16.

**Figure 17:**
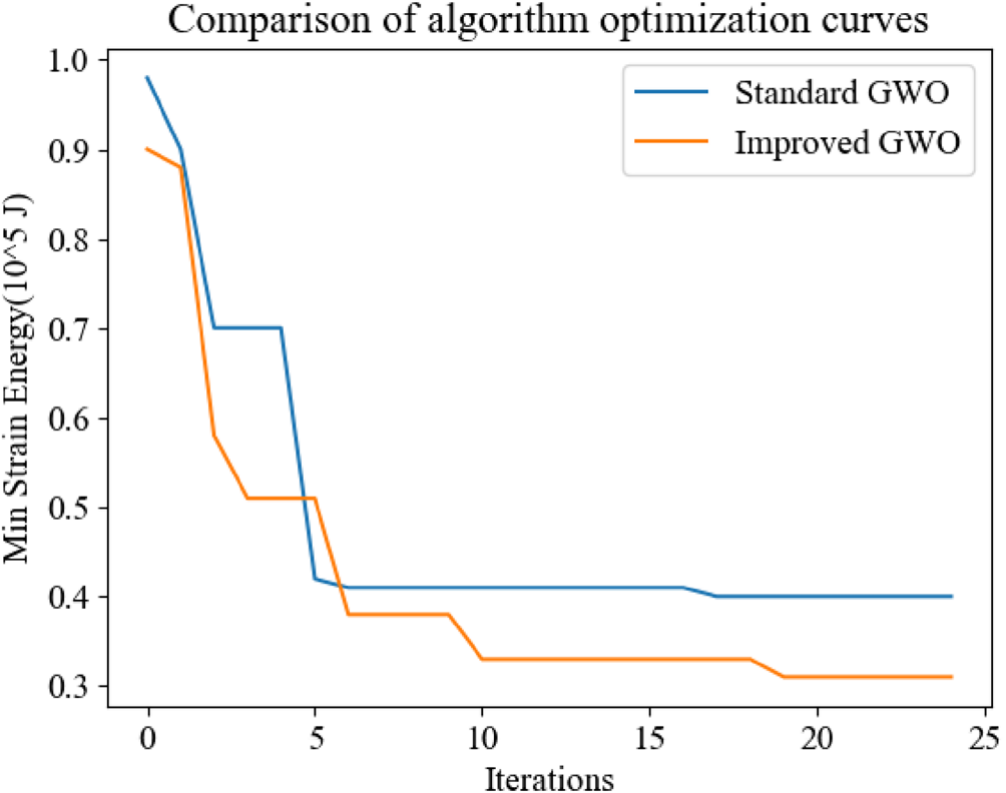
Comparison of optimization curves.

Firstly, the simulation experiment compares the optimization effect of the original GWO with the improved algorithm in this paper. The optimization effect is shown in Fig. 17.

**Figure 18:**
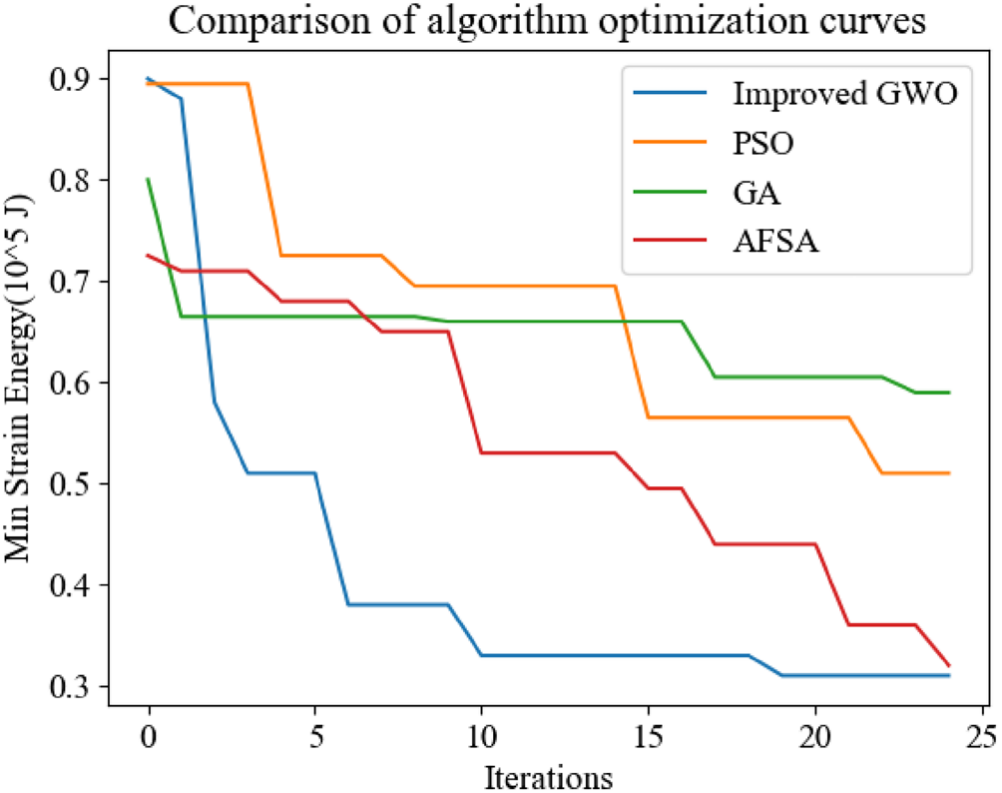
Optimization curves of improved algorithm and other optimization algorithms.

Through simulation, it can be seen that the improved GWO has stronger optimization ability and better optimization effect than the standard GWO, and converges faster in the four-point lifting location optimization problem, which makes the overall strain energy of transmission line tower lower and has obvious improvement effect. The optimization curve pairs of the improved GWO and other swarm intelligence optimization algorithms are shown in Fig. 18.

The experiment proves that the improved Gray Wolf optimization algorithm effectively avoids the algorithm falling into the local optimum and stagnating through the initialization method of the good point set, improves the global convergence, and optimizes the algorithm's optimization performance based on the combination of the variation adjustment strategy and the median optimization strategy. Compared with genetic algorithm and particle swarm optimization algorithm, the optimization effect is stronger, and it can find a better lifting point. Compared with the artificial fish swarm optimization algorithm, the convergence speed is faster in the same number of iterations, and the optimization effect is relatively better.

## Conclusion

In this paper, for the optimization problem of transmission tower lifting point position, three improvement methods are proposed to enhance the effect of the optimization algorithm by addressing the problems of easy to fall into local minima, and poor optimization effect in the current Gray Wolf optimization algorithm. The experimental results show that the improved algorithms proposed in this paper obtain good optimization results in several test functions, and have better optimization results in the transmission tower lifting point optimization problem than other commonly used swarm intelligence optimization algorithms, but there is a lack of generalization experiments to apply the improved algorithms to other engineering problems, and the improved algorithms may suffer from a decrease in the performance of optimization in the face of a more complex problem. The problem is that the performance of the improved algorithm may be degraded when facing more complex problems. Therefore, in the future research, we will study the robustness and generalization of GWO in different engineering problems to further improve the performance of the algorithm.

## Data Availability

The data used to support the findings of this study are available from the corresponding author upon request.
